# Blockade of Airway Inflammation by Kaempferol via Disturbing Tyk-STAT Signaling in Airway Epithelial Cells and in Asthmatic Mice

**DOI:** 10.1155/2013/250725

**Published:** 2013-05-08

**Authors:** Ju-Hyun Gong, Daekeun Shin, Seon-Young Han, Sin-Hye Park, Min-Kyung Kang, Jung-Lye Kim, Young-Hee Kang

**Affiliations:** Department of Food and Nutrition, Hallym University, Chuncheon, Gangwon-do 200-702, Republic of Korea

## Abstract

Asthma is characterized by bronchial inflammation causing increased airway hyperresponsiveness and eosinophilia. The interaction between airway epithelium and inflammatory mediators plays a key role in the asthmatic pathogenesis. The *in vitro* study elucidated inhibitory effects of kaempferol, a flavonoid found in apples and many berries, on inflammation in human airway epithelial BEAS-2B cells. Nontoxic kaempferol at ≤20 **μ**M suppressed the LPS-induced IL-8 production through the TLR4 activation, inhibiting eotaxin-1 induction. The *in vivo* study explored the demoting effects of kaempferol on asthmatic inflammation in BALB/c mice sensitized with ovalbumin (OVA). Mouse macrophage inflammatory protein-2 production and CXCR2 expression were upregulated in OVA-challenged mice, which was attenuated by oral administration of ≥10 mg/kg kaempferol. Kaempferol allayed the airway tissue levels of eotaxin-1 and eotaxin receptor CCR3 enhanced by OVA challenge. This study further explored the blockade of Tyk-STAT signaling by kaempferol in both LPS-stimulated BEAS-2B cells and OVA-challenged mice. LPS activated Tyk2 responsible for eotaxin-1 induction, while kaempferol dose-dependently inhibited LPS- or IL-8-inflamed Tyk2 activation. Similar inhibition of Tyk2 activation by kaempferol was observed in OVA-induced mice. Additionally, LPS stimulated the activation of STAT1/3 signaling concomitant with downregulated expression of Tyk-inhibiting SOCS3. In contrast, kaempferol encumbered STAT1/3 signaling with restoration of SOCS3 expression. Consistently, oral administration of kaempferol blocked STAT3 transactivation elevated by OVA challenge. These results demonstrate that kaempferol alleviated airway inflammation through modulating Tyk2-STAT1/3 signaling responsive to IL-8 in endotoxin-exposed airway epithelium and in asthmatic mice. Therefore, kaempferol may be a therapeutic agent targeting asthmatic diseases.

## 1. Introduction

Allergic asthma is characterized by the infiltration of eosinophils, mast cells, and T-lymphocytes into airway epithelium [1, 2]. This infiltration usually leads to bronchial epithelial layer desquamation, goblet cell hyperplasia, and submucosa thickening [3]. The interplay between airway epithelial cells and the immune cells plays an important role in the pathogenesis of an allergic asthma attack [4]. Accordingly, the airway epithelium is both a target of inflammatory and physical insults and an effecter of ongoing airway inflammation. In asthmatic process, antigen-sensitized T helper 2 (Th2) cells produce specific cytokines, which cause several key features of allergic bronchial asthma [5]. Both IL-4 and IL-13 may stimulate epithelial cells to produce chemokines such as eotaxin and growth factors [6]. The eosinophil attachment and infiltration into the airway epithelium entail binding of eotaxin to C-C chemokine receptor type 3 (CCR3) expressed on eosinophils [2]. Proinflammatory IL-8 is secreted by macrophages and lung epithelial cell into lung fluid and recruits neutrophils and eosinophils to the sites of inflammation [7]. Accordingly, the IL-8 overexpression in human bronchial epithelial cells may play a pivotal role in the eosinophil infiltration into inflamed airways [8].

Exposure to lipopolysaccharide (LPS) increases the severity of asthma, which activates Toll-like receptor (TLR) signaling in the regulation of Th2-driven lung inflammation [9]. Several studies have shown that the TLR4 activation by LPS promotes inflammatory mechanisms including nuclear factor (NF)-*κ*B and Janus-activated kinase (JAK)/signal transducers and activators of transcription (STAT) pathways [10]. Cytokine stimulation activates the STAT pathway via phosphorylation of tyrosine residues by receptor-associated JAK family members [11]. Thus, the regulation of IL-8 response in airway epithelial cells through the inflammatory signaling pathway prevents explosive inflammatory reactions. The suppressors of cytokine signaling (SOCS) have emerged as the physiological or pathological regulators of cytokine responses in the inflammatory systems [12]. The SOCS proteins have important mechanism for the negative regulation of the cytokine-STAT pathway [13]. STAT6 is important in the regulation of lung inflammation in response to allergens and viruses in murine models with asthma [14]. However, much less is known about the role of STAT1/3 in mediating allergic responses in asthma.

Kaempferol is a natural flavonol-type flavonoid that has been isolated from plant sources. Kaempferol effectively suppresses the development of IgE-mediated allergic inflammation of intestinal cell models by inhibiting the secretion of allergic mediators [15]. The flavonol fisetin ameliorates asthmatic phenotypes, which is associated with reduction of Th2 responses as well as suppression of NF-*κ*B and its downstream chemokines [16]. Quercetin and kaempferol inhibited IgE-mediated release of proinflammatory mediators from human mast cells, which may be due to inhibition of intracellular calcium influx and PKC*θ* signaling [17]. Recently, we have demonstrated that kaempferol suppresses eosinophil infiltration and airway inflammation in allergic asthma [18]. It was also found that kaempferol attenuated airway allergic responses through disturbing NF-*κ*B signaling and eotaxin-1 secretion. However, the molecular mechanisms underlying the antiallergic actions of kaempferol should be fully clarified.

Based on the literature evidence that kaempferol possesses anti-inflammatory and antiallergic activities, this study investigated whether kaempferol inhibited inflammation in LPS-induced airway epithelial BEAS-2B cells through blocking TLR4 activation. It was tested that IL-8 was responsible for LPS-stimulated eotaxin-1 induction in epithelial cells. Furthermore, the suppressive effects of kaempferol on airway inflammation were evaluated in OVA-challenged BALB/c mice by measuring macrophage inflammatory protein (MIP)-2, CCR3, and eotaxin-1. This study elucidated whether kaempferol encumbered Tyk-STAT signaling pathway responsive to LPS and OVA in airway inflammation and eosinophilia.

## 2. Materials and Methods

### 2.1. Chemicals

M199, human epidermal growth factor (EGF), hydrocortisone, gelatin, human insulin, apotransferrin, LPS, and albumin from chicken egg white were obtained from the Sigma-Aldrich Chemical (St. Louis, MO, USA), as were all other reagents, unless specifically stated elsewhere. Fetal bovine serum (FBS), penicillin-streptomycin, and trypsin-EDTA were purchased from the Lonza (Walkersville, MD, USA). Human bronchial airway epithelial cell line, BEAS-2B, was provided by the American Type Culture Collection (ATCC, Manassas, VA, USA). Imject Alum (aqueous solution of 40 mg/mL aluminum hydroxide and 40 mg/mL magnesium hydroxide plus inactive stabilizers) was purchased from Thermo Fisher Scientific (Rockford, IL, USA). For western blot analysis and immunohistochemical assay, antibodies against human phospho-Tyk2, human phospho-STAT1/3, STAT3, and mouse phospho-STAT3 were obtained from Cell Signaling Technology (Beverly, MA, USA). Antihuman eotaxin-1 and antihuman IL-8 were purchased from R&D Systems (Minneapolis, MN, USA). Antihuman TLR4, antimouse CCR3, and antihuman SOCS3 were purchased from the Santa Cruz Biotechnology (Santa Cruz, CA, USA). Human Tyk2 inhibitor was provided by Calbiochem (Darmstadt, Germany). Horseradish peroxidase-conjugated goat antirabbit IgG, donkey antigoat IgG, and goat antimouse IgG were acquired from Jackson Immunoresearch Laboratories (West Grove, PA, USA). Albumin from bovine serum (essentially fatty acid free) and skim milk were acquired from Becton Dickinson Company (Sparks, MD, USA). Enzyme-linked immunosorbent assay (ELISA) kits of human IL-8, mouse MIP-2, and mouse eotaxin-1 were purchased from R&D Systems.

### 2.2. BEAS-2B Cell Culture and Viability

BEAS-2B cells were cultured in 25 mM HEPES-buffered M199 containing 10% FBS, 2 mM glutamine, 100 U/mL penicillin, 100 *μ*g/mL streptomycin supplemented with 2.5 *μ*g/mL insulin, 0.361 *μ*g/mL hydrocortisone, 2.5 *μ*g/mL apotransferrin, and 20 ng/mL human EGF. The 90–95% confluence of BEAS-2B cells was sustained at 37°C in an atmosphere of 5% CO_2_ during cell experiments. Kaempferol at 1–20 *μ*M was pretreated overnight, and then LPS or IL-8 applied to BEAS-2B cells to induce eotaxin-1, phospho-STAT1, and phospho-STAT3. A peak expression of eotaxin-1 was attained when LPS was added to BEAS-2B cells for 8 h [18].

The cytotoxicity of ≤20 *μ*M kaempferol was determined after 48 h culture of BEAS-2B cells using an MTT (3-(4,5-dimethylthiazol-yl)-diphenyl tetrazolium bromide, Duchefa Biochemie, Haarlem, The Netherlands) assay. Briefly, cells were maintained in a fresh medium including 1 mg/mL MTT at 37°C for 3 h. Gentle shaking was conducted to dissolve purple formazan product in 0.5 mL isopropanol, and the absorbance of formazan was determined at *λ* = 570 nm using a microplate reader (Bio-Rad Model 550, Hercules, CA, USA).

### 2.3. Induction of Airway Inflammation in a Murine Model

Six-week-old male BALB/c mice (Hallym University Breeding Center for Laboratory Animals) were kept on a 12 h light/12 h dark cycle at 23 ± 1°C with 50 ± 10% relative humidity under specific pathogen-free conditions. Mice were fed a nonpurified diet (RodFeedTM, DBL, Umsung, Korea) and were provided with water ad libitum at the Animal Facility of Hallym University. The nonpurified diet composition was as follows: not less than (NLT) 20.5% crude protein, NLT 3.5% crude fat, not more than (NMT) 8.0% crude fiber, NMT 8.0% crude ash, NLT 0.5% calcium, and NLT 0.5% phosphorus. Mice were allowed to acclimatize for 1 week before beginning the experiments. Mice were divided into four subgroups (*n* = 6 for each subgroup). Mice were sensitized with 20 *μ*g OVA dissolved in a solution of 30 *μ*L PBS and 50 *μ*L Imject Alum by subcutaneous injection twice on day 0 and day 14. Kaempferol solution (0.1 mL, 10 or 20 mg/kg BW) was orally administrated to OVA-sensitized mice 1 h before OVA challenge. On day 28, day 29, and day 30, the 5% OVA inhalation to mice was performed for 20 min in a plastic chamber linked to an ultrasonic nebulizer (Clenny^2^ Aerosol, Medel, S. Polo di Torrile, Italy). Control mice were sensitized and challenged with PBS as the OVA vehicle. All mice were sacrificed with an anesthetic (2 *μ*L/kg Rompun and 8 *μ*L/kg Zoletil, intraperitoneal injection) 24 h after the last challenge (day 30). The right lungs were collected, frozen to liquid nitrogen, and kept at −80°C for the extraction, and the left lungs were preserved and fixed in 4% paraformaldehyde and then used for the staining.

All experiments were approved by the Committee on Animal Experimentation of Hallym University and performed in compliance with the University's Guidelines for the Care and Use of Laboratory Animals (Hallym 2010-66). No mice were dead, and no apparent signs of exhaustion were observed during the experimental period.

### 2.4. Western Blot Analysis

Whole BEAS-2B cell lysates or BALB/c lung tissue extracts were prepared in 1 M Tris-HCl (pH 6.8) lysis buffer containing 10% SDS, 1% glycerophosphate, 0.1 M Na_3_VO_4_, 0.5 M NaF, and protease inhibitor cocktail. Equal volumes of cell culture supernatants and equal amounts of cell lysates or tissue extracts proteins were electrophoresed on 8–15% SDS-PAGE gel and transferred onto a nitrocellulose membrane. Blocking of nonspecific binding was performed in a TBS-T buffer (50 mM Tris-HCl (pH 7.5), 150 mM NaCl, and 0.1% Tween 20) containing either 3% bovine serum albumin or 5% nonfat dry milk for 3 h. The membrane was incubated overnight at 4°C with a specific primary antibody. After triple washing with TBS-T buffer, the membrane was then applied to a goat antirabbit IgG, donkey antigoat IgG, or goat antimouse IgG conjugated to horseradish peroxidase for 1 h. Following another triple washing, target protein was determined using the SuperSignal West Pico Chemiluminescence detection reagents (Pierce Biotechnology, Rockford, IL, USA) and the Agfa medical X-ray film blue (Agfa HealthCare NV, Mortsel, Belgium). Antihuman **β**-actin incubation was accomplished for the comparative control.

### 2.5. Reverse Transcriptase-Polymerase Chain Reaction (RT-PCR) Analysis

Following culture protocols, total RNA was isolated from LPS-treated BEAS-2B cells using a commercially available Trizol reagent kit (Invitrogen, Carlsbad, CA, USA). The RNA (5 *μ*g) was reversibly transcribed with 200 units of reverse transcriptase and 0.5 mg/mL oligo-(dT)_15_ primer (Bioneer, Daejeon, Republic of Korea). The expressions of the mRNA transcripts of TLR4 (forward primer 5′-CAT TGG TGT GTC GGT CCT CA-3′, reverse primer 5′-ACT GCC AGG TCT GAG CAA TC-3′, 377 bp) and **β**-actin (forward primer 5′-GAC TAC CTC ATG AAG ATC-3′, reverse primer 5′-GAT CCA CAT CTG CTG GAA-3′, 512 bp) were evaluated by RT-PCR. The PCR was performed in 25 *μ*L buffer (10 mM Tris-HCl (pH 9.0), 25 mM MgCl_2_, 10 mM dNTP, 5 units of Taq DNA polymerase, and 10 *μ*M of each primer) and terminated by heating at 94°C for 10 min. After thermocycling and electrophoresis of 25 *μ*L PCR products on 1.5% agarose-formaldehyde gel, the bands were visualized using a TFX-20 M model-UV transilluminator (Vilber-Lourmat, Marne-la-Vallée, France), and gel photographs were obtained. The absence of contaminants was routinely checked by the RT-PCR assay of negative control samples without a primer addition.

### 2.6. ELISA

Cell-free culture media were collected from BEAS-2B cells and stored at −20°C. IL-8 secretion and tissue levels of MIP-2 and eotaxin-1 were examined in culture media or BALB/c lung tissue extracts by using each ELISA kit (Microplate Reader, Molecular Devices, Sunnyvale, CA, USA).

### 2.7. Lung Immunohistochemistry

Immunohistochemical analysis was carried out by using antibodies against mouse CXCR2, mouse phospho-Tyk2, and phospho-STAT3. All lung tissue sections were subjected to a series of immunohistochemical procedures including the Ag retrieval followed by quenching of endogenous peroxidase activity. For the measurement of tissue levels of CXCR2 and phospho-Tyk2, an immunofluorescent histochemical analysis was conducted using antimouse CXCR2 or antimouse phospho-Tyk2 and Cy3- or FITC-conjugated antigoat IgG. Nuclear staining was done with 4′,6-diamidino-2-phenylindole (DAPI). For the detection of phospho-STAT3, 3,3′-diaminobenzidine chromogenic substrate detection kit (Dako, Carpinteria, CA, USA) was used. Counter staining was conducted with hematoxylin. Each slide was mounted in VectaMount mounting medium (Vector Laboratories, Burlingame, CA, USA). Images of each slide were taken using an optical microscope system (Axiomager, Zeiss, Germany). Protein levels of CXCR2, phospho-Tyk2, and phospho-STAT3 were quantified by the image analysis program of the microscope system.

### 2.8. Statistical Analysis

The data are presented as mean ± SEM for each treatment group in the *in vivo* and *in vitro* experiments. Statistical analyses were conducted using a Statistical Analysis Systems program (SAS Institute, Cary, NC, USA). One-way ANOVA was used to determine inhibitory effects of kaempferol on airway inflammation and allergic responses in epithelial cells and sensitized mice. Differences among treatment groups were analyzed with Duncan's multiple range test and were considered to be significant at *P* < 0.05.

## 3. Result

### 3.1. Suppression of LPS-Promoted TLR4 Induction and IL-8 Production by Kaempferol

**Figure 1:**
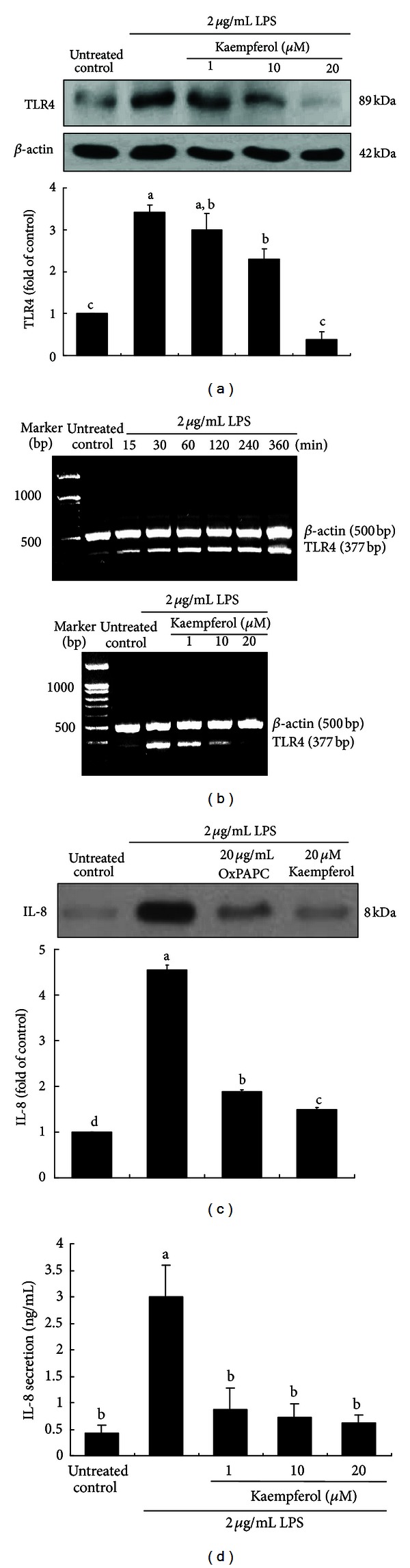
Inhibitory effects of kaempferol on expression levels (a) and transcription (b) of TLR4 and blockade of IL-8 secretion by TLR4 inhibition (c) and IL-8 secretion (d) in LPS-stimulated BEAS-2B cells. After culturing cells with 2 *μ*g/mL LPS in the absence and presence of 1–20 *μ*M kaempferol or 20 *μ*g/mL OxPAPC for 8 h, cell extracts were subjected to 8% SDS-PAGE and western blot analysis with a primary antibody against TLR4 and IL-8. Representative blot data were obtained from 3 experiments, and **β**-actin protein was used as an internal control. The mRNA levels of TLR4 were analyzed by using RT-PCR (b). **β**-actin was used as a housekeeping gene for the coamplification with TLR4. The bar graphs (mean ± SEM) in the bottom panels represent quantitative results of blots. Cell culture media were collected for the measurement of IL-8 secretion by using an ELISA kit (d). Values not sharing a common letter are significantly different at *P* < 0.05.

Mammalian TLR4 is the signal-transducing receptor activated by the bacterial LPS and lipotechoic acid [10, 19]. Western blot analysis showed that TLR4 served as an epithelial receptor to LPS for the airway inflammatory process. Human BEAS-2B cells were incubated with 2 *μ*g/mL LPS in the absence and presence of 1–20 *μ*M kaempferol for 8 h. The expression of TLR4 was greatly elevated in LPS-stimulated BEAS-2B cells (Figure 1(a)). This study investigated whether 1–20 *μ*M kaempferol inhibited the induction of TLR4 triggered by LPS. When BEAS-2B cells were incubated with ≤20 *μ*M kaempferol for 24 h, there was no notable cytotoxicity observed [18]. When nontoxic kaempferol was added, the TLR4 induction was inhibited in a dose-dependent manner. Additionally, kaempferol suppressed the expression of TLR4 mRNA (Figure 1(b)).

This study elucidated that LPS induced cellular expression of IL-8 through stimulating TLR4 signaling and that kaempferol encumbered IL-8 induction. LPS enhanced cellular secretion of IL-8, which was dampened by the nontoxic TLR inhibitor OxPAPC at 20 *μ*g/mL (Figure 1(c)). Similar inhibition was observed with 20 *μ*M kaempferol. In addition, LPS increased the IL-8 secretion of BEAS-2B cells (Figure 1(d)). However, the treatment of LPS-exposed cells with ≥1 *μ*M kaempferol markedly attenuated such secretion.

**Figure 2:**
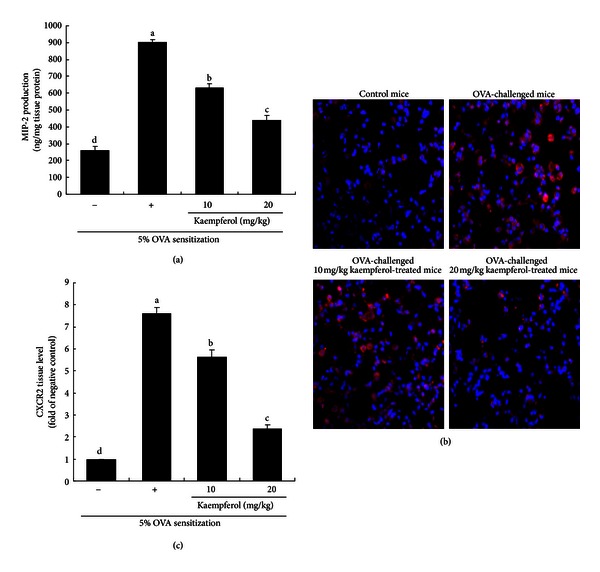
Inhibition of MIP-2 production (a) and CXCR2 expression (b and c) by kaempferol. Inhibition of MIP-2 production and CXCR2 expression by kaempferol in OVA-challenged mouse lung tissues. Tissue extracts were collected for the measurement of MIP-2 production by using an ELISA kit (a). Immunofluorescence analysis showing inhibition of CXCR2 expression in OVA-challenged mouse lung tissues by kaempferol (b). CXCR2 localization was visualized with a Cy3-conjugated secondary antibody. Nuclear staining was done with DAPI. CXCR2 was identified as red staining and quantified by using an optical microscope system (c). Each photograph was representative of four mice. Magnification: 200-fold. Values in the bar graphs (mean ± SEM) not sharing a common letter are significantly different at *P* < 0.05.

The current study attempted to prove that IL-8 is one of the pivotal factors responsible for asthmatic airway inflammation. Chemokines with protein sequence homology to human IL-8 have not been identified in mice [20]. The CXC chemokines KC and MIP-2 (also known as CXCL2) are functional homologs of human IL-8 in mice. Accordingly, the MIP-2 levels in mouse lung tissue were measured. OVA challenge increased MIP-2 production in mouse lung tissue (Figure 2(a)). However, kaempferol supplemented to OVA-challenged mice markedly diminished MIP-2 production. Furthermore, this study examined the induction of CXCR2, the receptor to IL-8, in lung tissues of OVA-challenged mice. There was lack of cytoplasmic staining in the negative control mice (Figure 2(b)). However, a strong cytoplasmic reddish staining was observed in OVA-challenged mice. In contrast, the CXCR2 induction was dose dependently attenuated in mice supplemented with kaempferol (Figure 2(c)).

### 3.2. Attenuation of LPS-Induced Eotaxin-1 Expression by Kaempferol

**Figure 3:**
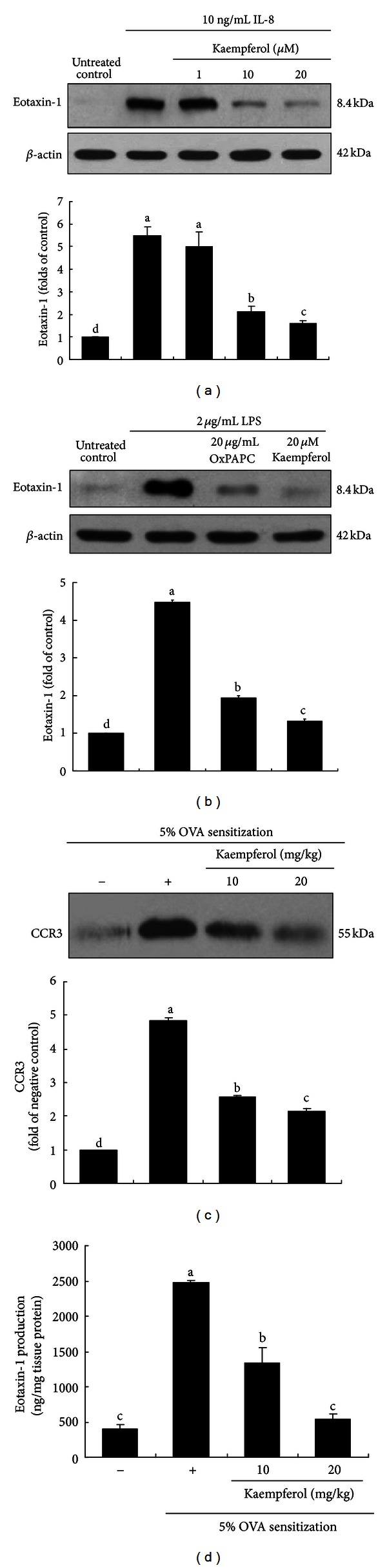
Western blot data showing inhibition of eotaxin-1 expression by kaempferol or OxPAPC in LPS- or IL-8-stimulated in BEAS-2B cells (a and b). After culturing cells with 2 *μ*g/mL LPS in the absence and presence of 1–20 *μ*M kaempferol or 20 *μ*g/mL OxPAPC for 8 h, cell extracts were subjected to 15% SDS-PAGE and western blot analysis with a primary antibody against eotaxin-1. **β**-actin protein was used as an internal control. Inhibition of CCR3 expression and eotaxin-1 secretion by kaempferol in OVA-challenged mouse lung tissues (c and d). Tissue extracts were subjected to western blot analysis with a primary antibody against murine CCR3 (c). The bar graphs (mean ± SEM, *n* = 3) in the bottom panels represent quantitative results. Eotaxin-1 production was measured in OVA-challenged mouse lung tissues by using an ELISA kit (d). Values in bar graphs not sharing a letter indicate significant difference at *P* < 0.05.

This study investigated whether IL-8 was involved in the eosinophil infiltration by inducing eotaxin-1 protein in endotoxin-experienced airway epithelial cells. Eotaxin-1 expression was greatly enhanced in IL-8-stimulated BEAS-2B cells, which was reversed by treating ≥10 *μ*M kaempferol (Figure 3(a)). Accordingly, the suppression of IL-8 production by kaempferol may be associated with its blockade of early airway inflammation. Additionally, 20 *μ*g/mL OxPAPC abolished the induction of eotaxin-1 protein in LPS-exposed BEAS-2B cells (Figure 3(b)), indicating that its induction by LPS was mediated via the TLR4 signaling encumbered by kaempferol.

The role of eotaxin-1 in the airway inflammation was verified in lung tissues of OVA-challenged mice. CCR3 can serve as a receptor for several different chemokines such as macrophage inflammatory proteins, monocyte chemoattractant proteins, and eotaxins.

Most of the ligands to CCR3 are associated with asthma, and CCR3 has become an appealing possibility in asthma treatment or therapy [21]. The lung tissue level of CCR3 was enhanced in OVA-exposed mice (Figure 3(c)). In contrast, the supplementation of kaempferol abrogated the CCR3 protein level at the kaempferol-given dosages of 10 and 20 mg/kg. Additionally, this study determined the eotaxin-1 production in lung tissues of OVA-challenged mice. OVA elevated the eotaxin-1 protein level in mouse lung tissues (Figure 3(d)). However, in OVA-experienced mice supplemented with kaempferol, the eotaxin-1 production was dose dependently diminished.

### 3.3. Inhibitory Effect of Kaempferol on Tyk2-STAT Activation

**Figure 4:**
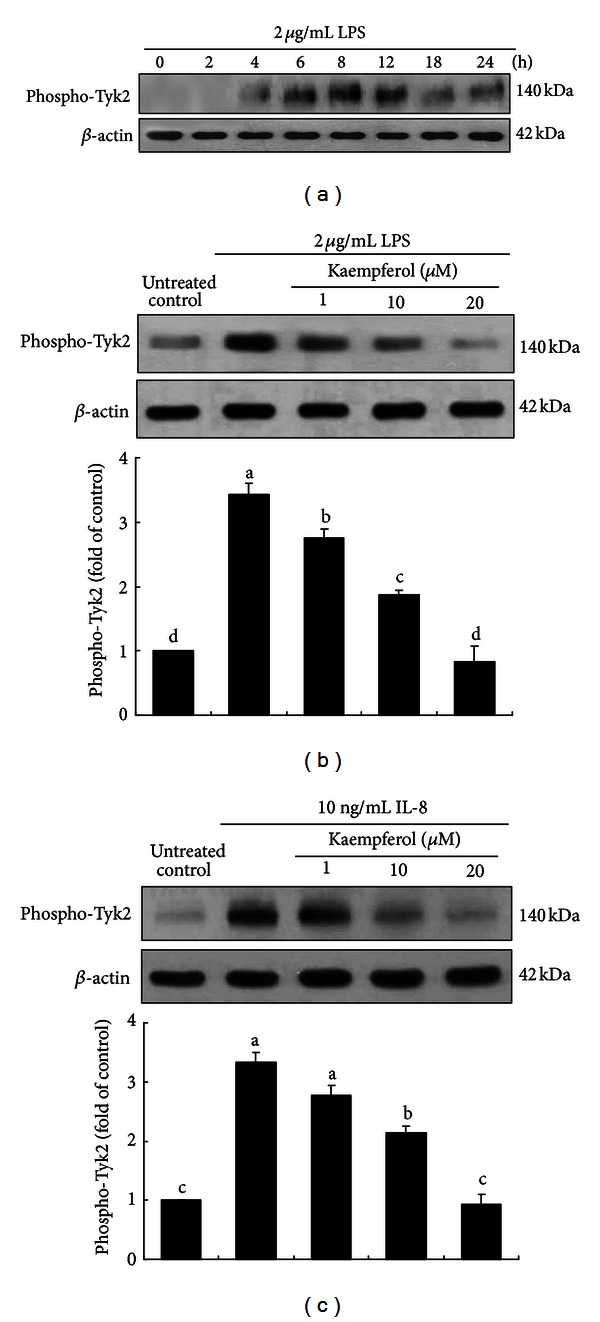
Temporal activation of Tyk2 (a) and its blockade by kaempferol in LPS-exposed epithelial cells. BEAS-2B cells were cultured with 2 *μ*g/mL LPS (b) or 10 ng/mL IL-8 (c) in the absence and presence of 1–20 *μ*M kaempferol for 8 h. Total cell protein extracts were subjected to western blot analysis with a primary antibody against phosphorylated Tyk2. **β**-actin was used as an internal control. The bar graphs (mean ± SEM, *n* = 4) in the bottom panels represent quantitative densitometric results. Means without a common letter differ, *P* < 0.05.

Activation of TLR4 by LPS leads to promotion of the inflammatory mechanisms including JNK/SAPK, NF-**κ**B, and JAK/STAT pathways [10]. This study elucidated whether Tyk downstream signaling was responsible for airway inflammation induced by LPS. BEAS-2B cells were incubated with 2 *μ*g/mL LPS, and Tyk2 activation was determined based on 2–6 h interval up to 24 h. Tyk2 phosphorylation was gradually elevated up to 8–12 h and thereafter diminished (Figure 4(a)). When kaempferol was added to LPS-exposed BEAS-2B cells, the Tyk2 activation was suppressed in a dose-dependent manner (Figure 4(b)). Similar effects on Tyk activation were observed with IL-8 (Figure 4(c)).

**Figure 5:**
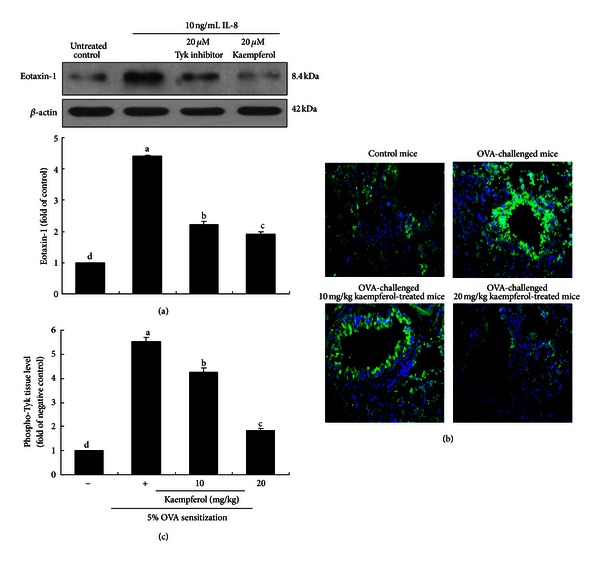
Effect of Tyk2 inhibition on eotaxin-1 expression in IL-8-stimulated BEAS-2B cells (a). BEAS-2B cells were treated with 20 *μ*M Tyk inhibitor and 20 *μ*M kaempferol exposed to 10 ng/mL IL-8 for 8 h. Cell extracts were subjected to western blot analysis with a primary antibody against eotaxin-1 (3 separate experiments, (a)). **β**-actin was used as an internal control. The bar graphs (mean ± SEM) in the bottom panel represent densitometric results. Immunofluorescence analysis showing inhibition of Tyk2 activation in OVA-challenged mouse lung tissues by kaempferol (b and c). Cytoplasmic Tyk2 was visualized with an FITC-conjugated secondary antibody. Nuclear staining was done with DAPI. Tyk2 was identified as green staining and quantified by using an optical microscope system (c). Each photograph is representative of four mice. Magnification: 200-fold. Means without a common letter differ, *P* < 0.05.

This study further tested whether the eotaxin-1 induction through TLR4 signaling by both LPS and IL-8 entailed Tyk2 activation. The Tyk inhibitor at 20 *μ*M suppressed the eotaxin-1 induction in IL-8-stimulated BEAS-2B cells in a similar manner to 20 *μ*M kaempferol (Figure 5(a)). Likewise, phosphorylated Tyk2 was notably observed in peribronchial regions of OVA-exposed mouse lung tissues, evidenced by immunofluorescent FITC tissue staining (Figure 5(b)). However, the FITC green fluorescence disappeared in lung tissues by supplying kaempferol to OVA-challenged mice at the dosages of 10 and 20 mg/kg (Figure 5(c)), indicating that kaempferol diminished airway inflammation by deterring the Tyk2 activation.

### 3.4. Disturbance of STAT3 Transactivation by Kaempferol

**Figure 6:**
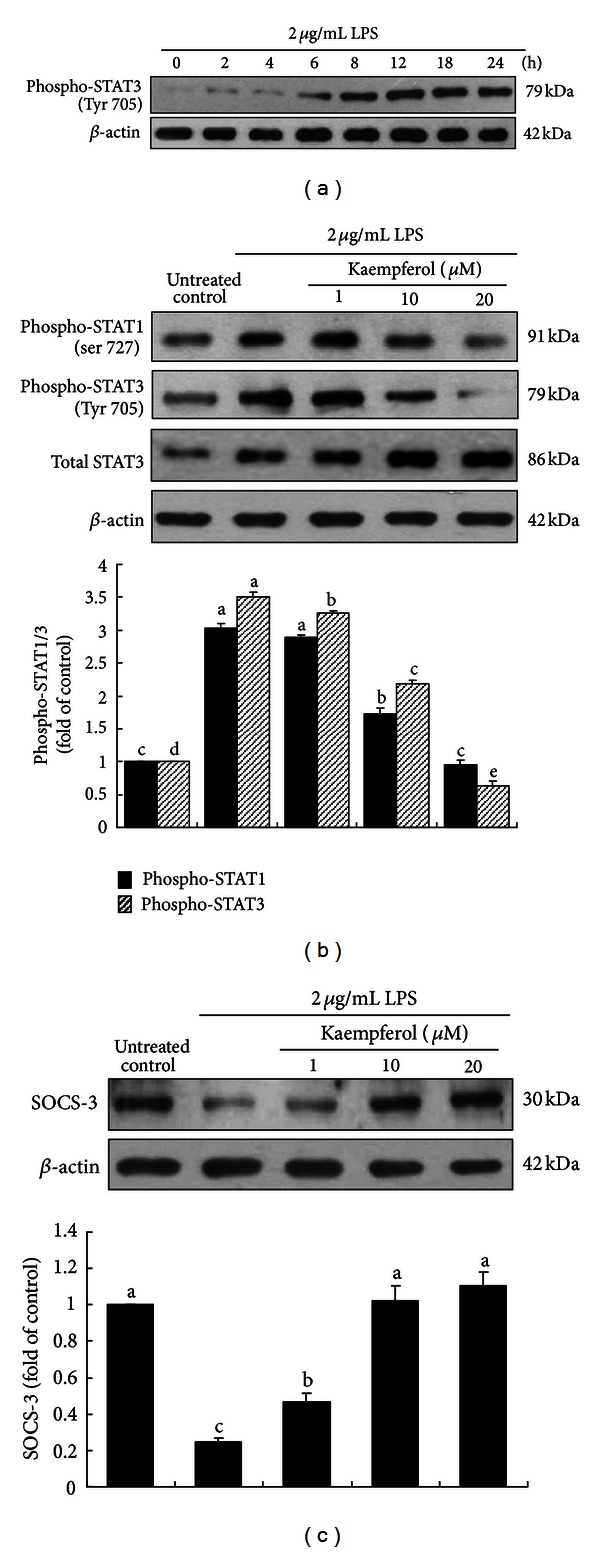
Time course response of STAT3 (a), suppression of STAT1/3 phosphorylation (b), and elevation of SOCS-3 expression (c) by kaempferol in LPS-stimulated BEAS-2B cells. BEAS-2B cells were treated with 1–20 *μ*M kaempferol in the absence and presence of 2 *μ*g/mL LPS for 8 h. For western blot analysis, total cell protein extracts were immunoblotted with a primary antibody against phosphorylated STAT1, phosphorylated STAT3, total STAT3, SOCS-3, or **β**-actin as an internal control. The bar graphs (mean ± SEM, *n* = 3) in the bottom panels represent quantitative densitometric results. Means without a common letter differ, *P* < 0.05.

Next, this study examined whether the phosphorylation of STAT1 and STAT3, the Tyk downstream effectors, was promoted by LPS in BEAS-2B cells. The phosphorylation of both STAT1 and STAT3 peaked at 8 h and stayed up in LPS-exposed BEAS-2B cells (Figure 6(a)). When BEAS-2B cells were activated by 2 *μ*g/mL LPS, ≥10 *μ*M kaempferol significantly suppressed the phosphorylation of STAT1 and STAT3, resulting in an increase in unphosphorylated STAT3 (Figure 6(b)). Thus, kaempferol may be an antagonist to this induction of STAT1/3 signaling in response to LPS in BEAS-2B cells. This implies that LPS promoted Tyk2 activation and sequentially activated STAT1/3 signaling leading to airway inflammation.

SOCS family members are cytokine-inducible negative regulators of cytokine signaling. The expression of SOCS3, the protein binding to Tyk2/JAK2 and inhibiting their activity, was dampened in LPS-experienced BEAS-2B cells (Figure 6(c)). This result proved that LPS positively regulated STAT signaling pathway, while kaempferol disturbed this pathway by restoring the SOCS3 expression in an opposite fashion. Accordingly, kaempferol may blunt IL-8 signaling by enhancing the inhibitory function of SOCS3 targeting Tyk2 activity.

**Figure 7:**
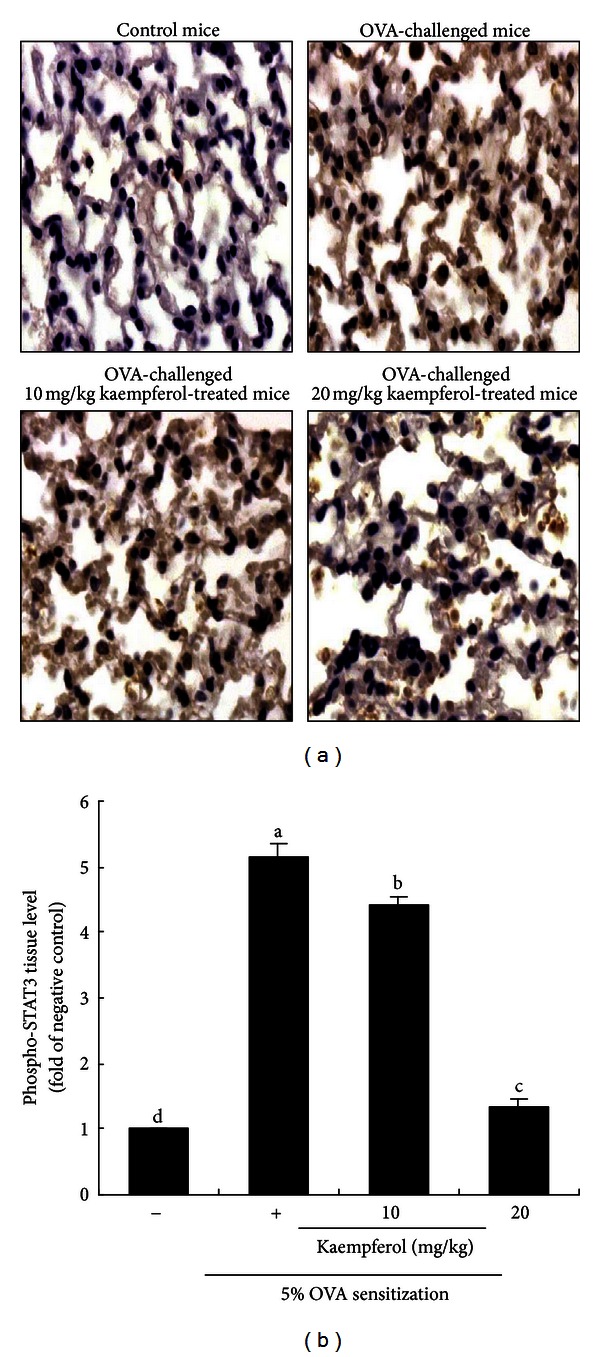
Immunohistochemical staining of phosphorylated STAT3 in lung tissues obtained from mice supplemented with 10–20 mg/kg kaempferol. Lung tissue sections were immunostained with a specific primary antibody against phosphorylated STAT3 and 3,3′-diaminobenzidine-conjugated IgG. Counter staining was conducted with hematoxylin. Phosphorylated STAT3 was identified as brown nuclear staining (a) and quantified by using an optical microscope system (b). Each photograph is representative of four mice. Magnification: 200-fold. Means without a common letter differ, *P* < 0.05.

Consistent with LPS-induced STAT3 activation in airway epithelial cells, the OVA challenge increased nuclear translocation of STAT3, manifested as brown nuclear staining. There was a significant increase in the numbers of dark brown-stained nuclear STAT3 observed (Figure 7(a)), demonstrating that the OVA incitement inflamed nuclear activation of STAT3. In contrast, the OVA-promoted STAT3 transactivation diminished in kaempferol-delivered mice (Figure 7(b)). Therefore, the specific blockade of Tyk-STAT in the airway/lung of sensitized mice by oral administration of kaempferol may be a useful anti-inflammatory strategy to confer asthmatic protection.

## 4. Discussion

Inflammatory and allergic asthma is characterized by the infiltration of eosinophils, mast cells, and T lymphocytes into airway epithelium [1, 2]. The interplay between these cells and airway epithelial cells plays an important role in the pathogenesis of an asthmatic episode [4]. The specific cytokines such as IL-4, IL-5, and IL-13 cause several key features of allergic bronchial asthma [5]. In addition, the eosinophil attachment and infiltration into the airway epithelium entail the binding of eotaxin proteins to CCR3 expressed on eosinophils, basophils, and Th2 cells [2]. The CXC chemokine IL-8 is a proinflammatory mediator associated with the chemotaxis and degranulation of neutrophils, T cells, basophils, and eosinophils [7]. It is produced by macrophages and lung epithelial cells into lung fluid and recruits neutrophils and eosinophils to the sites of inflammation. Accordingly, the overexpression of IL-8 in human bronchial epithelial cells plays a pivotal role in the recruitment and infiltration of eosinophils into inflamed airways, which lead to airway wall remodeling through activated intracellular signaling pathways. This study showed the enhanced secretion of IL-8 and eotaxin-1 from endotoxin-exposed airway epithelial cells and the increased induction of MIP-2 and CXCR2 in lung tissues of OVA-challenged mice. MIP-2 is a functional homolog of human IL-8 in mice. CXCR2 is the receptor to IL-8 that is a powerful neutrophil chemotactic factor. Also, kaempferol dampened epithelial secretion of IL-8 and eotaxin-1 and the induction of lung tissue CXCR2. Additionally, kaempferol inhibited the CCR3 induction and eotaxin-1 secretion enhanced by OVA challenge, indicating that kaempferol may inhibit inflammatory cell infiltration into the lesion sites of asthmatic inflammation. It should be noted that the eotaxin-1 secretion may be secondary to IL-8 induction.

Several polyphenols are effective in allaying allergic inflammation, resulting in symptom relief with the use of allopathic medicines [22–25]. Soy isoflavones suppress airway inflammation, hyperresponsiveness, and airway remodeling in a murine model of allergic asthma [23]. Chrysin inhibits mast cell-derived allergic inflammatory reactions by blocking production of histamine release and proinflammatory cytokines [24]. Although diverse undetermined molecular targets have been evidenced, the molecular mechanisms underlying antiallergic actions of polyphenols remain to be clarified [25, 26]. Our recent study demonstrated that kaempferol suppressed eosinophil infiltration and airway inflammation in allergic asthma through disturbing NF-*κ*B signaling and eotaxin-1 secretion [18]. Similarly, fisetin ameliorates asthmatic phenotypes concomitant with suppression of NF-*κ*B and its downstream chemokines [16]. In addition, casticin inhibits the eosinophil migration and activity of chemokines and adhesion molecules involved in the inflammatory process of asthma by suppressing the NF-*κ*B pathway [27]. Quercetin inhibits IgE-mediated release of proinflammatory mediators from human mast cells, possibly due to inhibition of intracellular calcium influx and PKC*θ* signaling [17]. However, the possible action mechanism(s) of kaempferol antagonizing the induction of inflammatory mediators responsible for airway allergic inflammation are not still defined.

Exposure to LPS increases the severity of asthma, which activates TLR signaling in regulation of Th2-driven airway disease [7]. In this study, the epithelial induction of IL-8 via TLR4 pathway stimulated eotaxin-1 expression associated with asthmatic inflammation. Consistently in OVA-challenged airway tissues MIP-2, CXCR2, and CCR3 were simultaneously induced, indicative of possible airway activation of eotaxin-1 by IL-8. Most of ligands to CCR3 are associated with asthma, and CCR3 has become an appealing possibility in asthma treatment or therapy [21, 28]. Kaempferol suppressed the induction of CXCR2 and CCR3 enhanced by OVA challenge. Activated TLR4 leads to the promotion of the inflammatory mechanisms including several downstream pathways of mitogen-activated protein kinasen, NF-*κ*B, and JAK/STAT [10]. The current study investigated a Tyk-STAT-responsive mechanism by which kaempferol disabled the IL-8 responses in lung/airway epithelial cells through inflammatory TLR4 signaling pathway. The downregulation of IL-8 response by kaempferol in airway epithelial cells through disturbing signaling pathways of Tyk2-STAT1/3 prevented explosive asthmatic reactions due to eotaxin-1 activation.

The STAT proteins, cytokine-inducible transcription factors, are crucial for cytokine signaling and the acute phase responses [11]. However, their role in mediating allergic responses in asthma is not well defined. One investigation showed that airway epithelial STAT3 was responsible for allergic inflammation by modulating Th2 cell recruitment and effector function in a murine model of chronic asthma [29]. Our study found that STAT1 and STAT3 might be involved in endotoxin-induced airway epithelial IL-8 signaling and subsequent eotaxin-1 activation. Likewise, the inhibition of STAT3 and STAT5 ameliorated experimental asthma by modulating lung CD11c(+) dendritic cells phenotype and function [30]. Thus, targeting STAT1 and STAT3 may provide the basis for a novel therapy for asthmatic inflammation [31]. In the current study, kaempferol attenuated the STAT activation through blocking the IL-8-Tyk2 pathway linked to epithelial TLR4 signaling inflamed by LPS. Consistently, kaempferol diminished the levels of STAT3 activated in OVA-challenged mouse airway/lung tissues. The polyphenol hesperidin-3′-O-methylether inhibits airway hyperresponsiveness in a murine model of asthma by decreasing the number of inflammatory cells and OVA-specific IgE levels in serum and BALF [32]. In addition, chlorogenic acid suppresses pulmonary eosinophilia, IgE production, and Th2-type cytokine production in an OVA-induced allergic asthma through inhibiting activation of STAT6 and JNK [33]. On the other hand, SOCS proteins have an important mechanism for the negative regulation of the cytokine-STAT pathway. Thus, SOCS proteins have been explored as targets for therapeutic strategies in allergic asthma [12]. In this study, the SOCS expression was reduced in LPS-exposed airway epithelial cells, which was reversed by the kaempferol treatment. Collectively, kaempferol boosted the inhibition of the Tyk2-STAT1/3 pathway responsible for the cytokine signaling of IL-8 and eventually regulated allergic asthma phenotype.

In summary, this study investigated the potential of kaempferol as targets for therapeutic strategies in endotoxin- or cytokine-associated airway inflammation. Nontoxic kaempferol suppressed LPS-induced production of IL-8 and subsequent induction of eotaxin-1. The IL-8 induction of eotaxin-1 entailed the mediation of the TLR4 signaling pathway that was blocked by kaempferol. Additionally, this compound disturbed the IL-8-Tyk2-STAT1/3 signaling with upregulation of SOCS in endotoxin-exposed airway epithelial cells. In the *in vivo* BALB/c mouse study, kaempferol administration blocked the induction of MIP-2, CXCR2, and CCR3 in airway/lung tissues elevated by OVA challenge. Moreover, kaempferol encumbered the OVA challenge-inflamed Tyk2-STAT3 activation. Therefore, kaempferol was effective in ameliorating allergic and inflammatory airway diseases through disturbing Tyk-STAT-responsive signaling pathway instigated by IL-8 advent in cellular or animal models of allergic asthma.

## Abbreviations

CCR3: C-C chemokine receptor type 3
CXCR2: C-X-C chemokine receptor type 2
JAK: Janus kinase
IL: Interleukin
LPS: Lipopolysaccharide
NF-*κ*B: Nuclear factor-*κ*B
OVA: Ovalbumin
PKC: Protein kinase
SOCS: Suppressors of cytokine signaling
STAT: Signal transducers and activators of transcription
Th2: T helper 2
TLR: Toll-like receptor
Tyk: Tyrosine kinase.
